# Estimation of relatedness among non-pedigreed Yakutian cryo-bank bulls using molecular data: implications for conservation and breed management

**DOI:** 10.1186/1297-9686-42-28

**Published:** 2010-07-13

**Authors:** Ilma Tapio, Miika Tapio, Meng-Hua Li, Ruslan Popov, Zoya Ivanova, Juha Kantanen

**Affiliations:** 1 Biotechnology and Food Research, MTT Agrifood Research Finland, Jokioinen, FI-31600 Finland; 2 Yakutian Research Institute of Agriculture, 677002 Yakutsk, Sakha, Russia

## Abstract

**Background:**

Yakutian cattle, the last remaining native cattle breed in Siberia, are well adapted to the extreme sub-arctic conditions. Nowadays only *ca*. 1200 purebred animals are left in Yakutia. The semen of six Yakutian bulls was stored in a cryo-bank without any pedigree documentation because of the traditional free herding style of the population.

**Methods:**

To clarify the genetic relatedness between these bulls and to provide recommendations to use their semen in future conservation and breed management programs, we have analysed 30 autosomal microsatellites and mitochondrial DNA sequences in 60 individuals including the six for which semen has been stored. Four relatedness estimators were calculated. In addition, we assessed the value of the cryo-bank bulls for the preservation of genetic variation of the contemporary Yakutian cattle by calculating allelic and gene diversity estimates and mean molecular coancestries.

**Results:**

On the basis of microsatellite variability, including the Yakutian cryo-bank bulls increases the allelic variation in the contemporary population by 3% and in the male subpopulation by 13%. In terms of the mean molecular coancestries, they are less related to the contemporary cow population than the breeding bulls and therefore could be used to reduce inbreeding in the living population. Although 30 loci are insufficient to resolve definitely their relatedness categories, the data suggest four pairs of cryo-bank bulls as possible half-sibs.

**Conclusions:**

Our results show that even relatively limited cryo-bank storage of semen can carry allelic variation through a bottleneck. We propose a breeding scheme based on the rotation of breeding females and the division of cryo-bank bulls into three groups. Thus, if molecular data (e.g. autosomal microsatellite genotypes) for the contemporary population are available and based on relatively small-scale laboratory analyses, it is possible to avoid serious mistakes in their use for breeding applications. The approach suggested here based on the use of Yakutian cryo-bank semen can be easily extended to cryo-bank materials of other animals in future breeding programs.

## Background

Yakutian cattle are the last remaining native cattle breed of the East Asian 'Turano-Mongolian' type of *Bos taurus *in Siberia. They are distributed in the north-eastern region of the Sakha Republic (Yakutia) of the Russian Federation [1-3]. These cattle possess a number of traits, such as solid trunk, short strong legs and long thick winter coat, which make them adapted to the extreme sub-arctic conditions. Moreover, efficient thermoregulation, quick formation of subcutaneous fatty tissue and low metabolic rates at low temperatures (even down to -60°C) allow them to survive in harsh environments under poor feed conditions (e.g. [3]). Ancestors of Yakutian cattle can be traced back to indigenous cattle in Siberia, which migrated with the Yakuts *ca*. 1,000 years ago from the southern Baikal region to the northern regions of the Lena and Yana rivers. Yakutian cattle were purebred until 1929 and, from then on, were subjected to extensive crossbreeding with productive breeds [2]. Consequently, only *ca*. 1200 purebred Yakutian cattle individuals remain in three villages in the district of Eveno-Bytantaisky, one village of Uluu-Syhyy and four different farms close to Yakutsk City [1]. Currently the population comprises only 525 breeding cows and 28 breeding bulls. Yakutian cattle are classified as an endangered breed by the Food and Agriculture Organization of the United Nations (FAO) [4]. However, recent studies in a continental context have suggested that this breed is highly interesting for the conservation of cattle genetic diversity [3,5]. There is a need to conserve the breed for future cattle breeding actions as well as for scientific and cultural purposes.

Maintaining genetic variability and avoiding inbreeding are of great importance in the management of small animal populations. Inbreeding has a negative effect on fitness, productivity and several other phenotypic traits [6]. Meanwhile, a reduction in gene and allele diversity might reduce a population's response to environmental changes or artificial selection in the future [7,8]. Thus, *ex situ *banking of embryos, oocytes and semen plays a fundamental role in the conservation and management of small farm animal breeds [9]. Storage of genetic material represents a reservoir of a breed's genetic diversity and could be used to re-establish a breed, if needed. The only genetic material stored *ex situ *for Yakutian cattle is the semen from six bulls collected between 1980 and 1986. However, because of the traditional free herding style of these cattle in summer pastures, where several bulls mate randomly within a herd, pedigree records of these six bulls are not available and, thus, the traditional pedigree-based control of inbreeding is impossible in a meaningful way.

In the absence of pedigree records, molecular data from autosomal, maternally inherited mitochondrial DNA (mtDNA) or from paternally inherited Y-chromosomal markers can be used to estimate relatedness between animals [10-12]. The widely applied statistical approaches to infer relatedness among individuals can be classified into two categories: one involves the explicit pedigree reconstruction among all individuals in the sample; and the other is based on the best pairwise relationship between two individuals at a time based on either relatedness estimation [13-15] or likelihood techniques [16,17]. The weakness of the pairwise methods is that they do not take into account information from the reference population and the difficulty in distinguishing among relationships with similar patterns of alleles (e.g. [18]). However, pedigree reconstruction methods have been applied mainly to the reconstruction of full-sib families [19].

Survival of the last native cattle breed in Siberia, Yakutian cattle, is important for the local human community as a source of food and income [1], but also because it presents extreme adaptive potentials of the cattle species in general. However, due to the small census size, Yakutian cattle require a careful management strategy. Long-term cryo-conservation of embryos and semen should be considered seriously as they represent a resource for ongoing breeding activities and a secure way of preserving genetic diversity within the breed, should the living population encounter problems. Although molecular measures of genetic relatedness do not necessarily agree exactly with the true relatedness coefficients calculated from the pedigree records (but see [20]), they are the best relatedness indicators in the absence of recorded pedigree information (e.g. [11]). Therefore, the specific goals of the current study were to estimate genetic relatedness among the six Yakutian cryo-bank bulls using pairwise and pedigree reconstruction methods based on the analysis of autosomal microsatellites and mtDNA sequences. We have also assessed how much genetic variation such a limited *ex situ *bank could add to the contemporary population of Yakutian cattle. Our aim was to solve a practical conservation problem in a highly valued cattle breed and to see how helpful basic population genetics analyses are in solving such a breed management question. Our results also provide recommendations for future conservation and use of the six cryo-bank semen.

## Methods

### Sampling and data extraction

Genomic DNA was extracted from the frozen semen samples of six Yakutian cattle cryo-bank bulls (named Keskil, Moxsogol, Radzu, Erel, Sarial and Alii), whose semen had been stored for more than 20 years, according to the method described by [21]. For the genetic diversity comparison, a reference population consisting of 54 randomly sampled Yakutian cattle individuals from the State farm in the village of Kustur (17 individuals) and from private farms in the villages of Batagai-Alyta (17), Kustur (4) and Uluu-Syhyy (16) in the Sakha Republic were also included in the analysis [3]. The reference population included samples of 37 cows and 17 bulls, referred hereafter to as 'the cow subpopulation' and 'the bull subpopulation', respectively. Genotypes of the reference population using the same set of 30 autosomal microsatellites were obtained from a previous study by Li et al. [3].

### Molecular analysis

To determine the levels of mtDNA variability, DNA samples of the six Yakutian cryo-bank bulls were sequenced for a 375-nucleotide fragment of the mtDNA control region using the primers published in [22]. The sequenced fragment covers bases 15,960 to 16,334 as compared to the complete cattle mtDNA sequence (NC006853). Standard double-stranded sequencing was performed with DYEnamic ET Terminator Kit (Amersham Biosciences) using the primers for polymerase chain reaction (PCR) and 10 μL of purified PCR-product on a MegaBACE™ 500 DNA Sequencer (Amersham Biosciences). Complementary sequences were combined using the SEQUENCHER v4.6 software (Gene Codes Co, Ann Arbor, MI, USA). In addition, sequences of 24 random individuals from the reference population (accession numbers FJ014247-FJ014270) were obtained from a recent study [23]. The six Yakutian cryo-bank bulls and international reference animals were genotyped for the same set of 30 microsatellites (Table 1) as described in [3]. Information on primers and PCR conditions can be found in the Cattle Diversity Database http://www.projects.roslin.ac.uk/cdiv/markers.html.

**Table 1:** Information on microsatellite markers

Locus	BTA	*A*_O_	PIC	*P*
BM1824	1	4	0.39	0.547
BM2113	2	6	0.58	0.834
ETH10	5	5	0.39	0.543
ETH225	9	5	0.63	0.355
ETH3	19	4	0.60	0.908
HEL5	21	5	0.62	0.097
ILSTS005	10	3	0.32	0.813
INRA023	3	5	0.52	0.879
INRA035	16	3	0.18	0.002
INRA005	12	4	0.61	0.652
INRA063	18	3	0.33	0.407
BM1818	23	2	0.33	0.089
CSSM66	14	8	0.65	1.000
ETH152	5	4	0.51	0.969
HEL1	15	5	0.57	0.200
HEL13	11	4	0.44	0.143
HEL9	8	6	0.62	0.056
ILSTS006	7	6	0.67	0.115
INRA032	11	3	0.45	0.103
INRA037	11	5	0.52	0.265
TGLA227	18	8	0.68	0.421
TGLA126	20	4	0.68	0.216
TGLA122	21	6	0.67	0.745
HAUT24	22	4	0.50	0.747
HAUT27	26	8	0.69	0.389
CSRM60	10	7	0.62	0.206
MM12	9	4	0.51	0.181
ETH185	17	5	0.61	0.328
TGLA53	16	10	0.67	0.134
SPS115	15	4	0.41	0.264

Mean	-	5	0.53	-

For each locus are given the chromosomal location (BTA), and the summary statistics per locus such as the number of observed alleles (*A*_O_), polymorphism information content (PIC) and *P*-values for the deviation from Hardy-Weinberg equilibrium across the total 60 Yakutian samples

### Statistical analysis

To characterise the maternal lineages, multiple alignments of mtDNA sequences were performed using ClustalX version 1.81 [24]. The size of the aligned mtDNA control region fragment was 255 nucleotides between bases 16,021 and 16,275 compared to the complete cattle mtDNA sequence (NC006853). The number of haplotypes was estimated and pairwise genetic distances between haplotypes were calculated based on the number of nucleotide differences using MEGA version 3.1 [25].

Genetic variability of the autosomal microsatellite loci in the whole Yakutian cattle sample (60 individuals) was quantified by the observed number of alleles (*A*_O_) and polymorphism information content (PIC) per locus using the program Microsatellite TOOLKIT [26]. Locus-wise tests for Hardy-Weinberg equilibrium (HWE) due to heterozygote deficiency were performed with 10,000 Monte Carlo randomisations [27] and the 'U' statistic test [28] as implemented in the programs GENEPOP version 4.0 [29] and ML-Relate [17], respectively. The program GENEPOP was also used in the Fisher's exact tests for genotypic linkage disequilibrium (LD) between all pairs of microsatellites with a Markov chain method of 50,000 iterations and 100 batches.

Relationships among the six Yakutian cryo-bank bulls were estimated with the pairwise relatedness estimators, *r*_W _[15] and *r*_QG _[13], using the program SPAGeDi version 1.2 [30]. The calculation was based on autosomal microsatellite genotypes in all 60 individuals. Furthermore, pairwise relationships between the bulls were calculated with the maximum-likelihood estimator *r*_K _using the program ML-Relate [17]. Performances of *r*_W_, *r*_QG_, and *r*_K _were evaluated using a simulation approach as implemented in PEDAGOG [31]. Allele frequencies of the 30 microsatellites obtained from all 60 individuals were used as input data. Distribution of pairwise relatedness (*R*) estimates for each of the four simulated relationship categories [unrelated (UR), half-sibs (HS), full-sibs (FS), and parent-offspring (PO)] was based on the simulated genotypes from 1000 individual-pairs each. The sampling variance was calculated as the standard deviation of the mean *R *estimate for each simulation category separately. The bias among estimators was tested by comparing the mean and the expected *R *values (UR 0.0; HS 0.25; FS and PO 0.5). Two-tailed *t*-tests were used to evaluate the significance of potential bias. Critical significance values were adjusted for multiple tests with sequential Bonferroni correction.

Pedigree reconstruction among all individuals in the sample was performed using PARENTAGE version 1.0 [32]. Two chains with burn-in of 200 iterations, thinning of 400 and 2000 samples were applied. A Dirichlet prior for the allele frequencies was used and the prior for the distribution of offspring between males and females was set to be gamma (1, 2). Influence of the six Yakutian cryo-bank bulls on the genetic variability of the reference population and the bull subpopulation were investigated by calculating basic statistics such as gene and allelic diversities.

Molecular coancestry is similar to the genealogical coancestry coefficient [33] but is defined as the probability that two alleles taken at random, one from each individual, are identical by state. To test if the six Yakutian cryo-bank bulls were less related to the cow subpopulation (37 cows) than the bull subpopulation (17 bulls), we used the program MOL_COANC version 1.0 [34] to calculate the mean molecular coancestry for the whole Yakutian cattle population (60 individuals), for the all the bulls (23 bulls comprising the six cryo-bank bulls and the 17 reference bulls), for the 17 reference bulls, for six Yakutian cryo-bank bulls and for each of the 23 bulls separately. Mean molecular coancestry [33] between each bull and every cow was also calculated. The difference between the bull subpopulation (17 reference bulls) and the group of six Yakutian cryo-bank bulls was tested using a two-sample permutation test by the Hothorn and Hornik exactRankTests version 0.8-12 package for the *R *language.

## Results

### Mitochondrial data

Screening of the 255 nt fragment of the mtDNA control region identified 11 haplotypes defined by 17 variable sites that belong to the taurine mtDNA sub-haplogroups T2, T3 and T4 (Additional file 1) [35,36]. Six haplotypes were individual-specific, three haplotypes were shared by two samples, one haplotype was shared by four samples and the most common haplotype was shared by 14 individuals. MtDNA sequences of the six Yakutian cryo-bank bulls (accession numbers FJ014464-FJ014469) were characterized by six different haplotypes, four of which were not observed in the contemporary samples (Additional file 1). The average number of pairwise nucleotide differences among all 11 haplotypes was 3.78, ranging from 1 to 8 among pairs of comparison. The number of pairwise nucleotide differences among the six haplotypes observed in the six Yakutian cryo-bank bulls varied from 2 to 7 with an average number of 4.53. We did not find any mtDNA haplotype shared by all six Yakutian cryo-bank bulls, which indicates that these bulls cannot be full-sibs or maternal half-sibs.

### Microsatellites and relatedness

One hundred and fifty alleles were detected in the 60 Yakutian cattle individuals across the 30 microsatellites. The number of observed alleles varied from 2 to 10 per locus (Table 1). The average PIC across the loci for the complete sample was 0.532, with the lowest PIC observed at *INRA035 *(0.176) and the highest at *HAUT27 *(0.685). No significant (*P *< 0.05) deviations from LE were observed in the pairwise microsatellite comparisons after sequential Bonferroni correction was applied. Significant (*P *< 0.05) heterozygote deficiency was detected only at *INRA035 *(Table 1), which is probably due to the presence of non-amplifying alleles (e.g. [37]). It is also possible that the locus *INRA035 *is near a gene or within a genomic region under directional selection and this would be interesting to investigate further.

We calculated pairwise relatedness estimates between the six Yakutian cryo-bank bulls with and without the locus *INRA035*. These calculations of relatedness were further adjusted to accommodate non-amplifying alleles by the option as implemented in the ML-Relate program. Neither the exclusion of the locus nor the inclusion of the non-amplifying alleles had a significant effect on the relatedness estimates (not shown). Therefore, the results presented in the study are based on the full set of 30 microsatellites (Additional file 2).

Mean *r*_W _and *r*_QG _estimates and their standard deviations calculated for four simulated relatedness distributions are presented in Additional file 3. Performances of both pairwise relatedness estimators were similar to each other with only minor differences in variance estimates. *r*_QG _had a slightly smaller (by 0.004) sampling variance for the distribution of UR individuals, while *r*_W _performed better in the remaining categories (HS by 0.002, FS by 0.01 and PO by 0.015). In three out of eight cases, mean *R *deviated significantly from the expected value (*P *< 0.013 after sequential Bonferroni correction). The bias for *r*_W _for UN pairs was downwards, while that for *r*_W _and *r*_QG _in the category of PO was upwards (Additional file 3). The performance of *r*_K _was very similar to that of *r*_W _(results not shown) apart from negative values being converted to zero relatedness.

**Figure 1:**
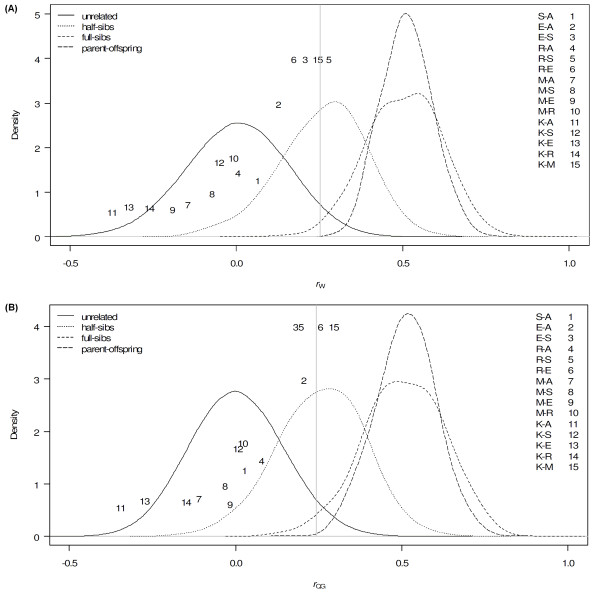
**Pairwise relatedness of Yakutian cryo-bank bulls**. Values are calculated using **(A) ***r*_W _and (**B) ***r*_QG _relatedness estimators plotted on a distribution of four simulated relationship categories: unrelated, half-sibs, full-sibs and parent-offspring; the vertical line represents the 95^th ^percentile for simulated unrelated individuals; the position of pairwise values in regards to the Y-axis was designed based on the estimates from the *r*_K _relatedness estimator and was calculated as 3 divided by the cases when the log likelihood of *R *for the second closest relationship is smaller than the most likely relationship; abbreviations for Yakutian cryo-bank bulls are: K-Keskil, M-Moxsogol, R-Radzu, E-Erel, S-Sarial, A-Alii. Y-axis denotes the distribution of posterior probability density based on the simulations of the four relationship categories using the two relatedness estimators *r*_W _and *r*_QG_, respectively.

Ten out of 15 pairwise *R*-estimates between the six Yakutian cryo-bank bulls approached zero or fell below it. The remaining five bull-pairs exhibited *R*-values ranging from 0.124 to 0.276 for *r*_W _and from 0.180 to 0.295 for *r*_QG _(Additional file 2). All pairwise *R *values were plotted on the distribution of four simulated relatedness categories (Figure 1). When the *r*_W _estimator was used, one pair (Radzu:Sarial, *R *= 0.276) fell outside the 95% confidence interval for simulated UR individuals (the 95^th ^upper quantile = 0.252) and was considered to be related (Figure 1a). Two other pairs were identified as related when the *r*_QG _estimator was applied (Keskil:Moxsogol, *R *= 0.295; Radzu:Erel, *R *= 0.255; the 95^th ^upper quantile = 0.242) (Figure 1b). The ML-Relate program uses simulation to determine which relationships are consistent with genotype data and to compare putative relationships with alternatives. In order to identify possible misclassified individuals, a maximum-likelihood estimator *r*_K _estimated by ML-Relate was applied. Besides the three bull-pairs mentioned above, the Erel:Sarial pair (*r*_W _= 0.205; *r*_QG _= 0.180) had the highest likelihood of being a half-sib (Additional file 2). The same four pairs of Yakutian cryo-bank bulls were also identified as potential half-sibs in the parentage analysis performed using the pedigree reconstruction method among all individuals in the sample (Additional file 4).

### Allelic diversity and gene diversity

Inclusion of the six Yakutian cryo-bank bulls in the calculation increases the within-population genetic variability relative to that in the contemporary reference population (Table 2). For example, the six cryo-bank samples made it possible to add two new alleles at the locus *INRA023 *and their frequency in the cryo-bank samples is 0.083. Therefore, compared to the three alleles detected in the 54 contemporary samples from the reference population, a 67% gain in allelic variation was observed when including the six cryo-bank samples. With the six cryo-bank bulls, the average allelic diversity of the total Yakutian population increased by 3%, while the average allelic diversity of the bulls increased by 13%. Frequencies of alleles specific for the cryo-bank bulls ranged from 0.083 to 0.250. Three Yakutian cryo-bank bulls, Keskil, Radzu and Alii, carried alleles not detected in the contemporary Yakutian population. Furthermore, all six Yakutian cryo-bank bulls possessed microsatellite alleles that were not found in the contemporary bull subpopulation. The gene diversity would increase by 3.5% if the six cryo-bank bulls represented the total bull subpopulation in the next generation together with the contemporary cows. The increase in gene diversity would be 1.2% by adding cryo-bank bulls to the contemporary bull subpopulation in the calculation.

**Table 2:** Increase in allelic variation when the six Yakutian cryo-bank bulls are included in the analysis

	Total (*N *= 60)				Males (*N *= 23)			
	***N*_A_**	**%**	**Frequency**	**Individual**	***N*_A_**	**%**	**Frequency**	**Individual**

ETH10	0	0	-		1	25	0.167	Keskil, Erel
HEL5	0	0	-		1	25	0.083	Sarial
INRA023	2	67	0.083	Radzu, Alii	2	67	0.083	Radzu, Alii
INRA063	0	0	-		1	50	0.250	Keskil, Moxsogol, Sarial
CSSM66	0	0	-		1	17	0.167	Erel
ILSTS006	0	0	-		2	67	0.250	Keskil, Moxsogol
TGLA227	1	14	0.083	Keskil	1	17	0.083	Keskil
TGLA122	0	0	-		2	50	0.083	Moxsogol, Erel
HAUT27	0	0	-		1	14	0.083	Keskil
ETH185	0	0	-		1	25	0.167	Keskil, Moxsogol
TGLA53	1	11	0.083	Keskil	1	17	0.083	Keskil
SPS115	0	0	-		1	50	0.083	Alii

Average		3				13		

The number (*N*_A_) and frequency of added alleles in the six cryo-bank samples, the percentage gain in allelic variation (%), and the name of the Yakutian cryo-bank bulls contributing new alleles to the population are indicated when all the samples (54 + 6 individuals) and the bull samples (17 + 6 individuals) are considered; the percentage gain in allelic variation (%) was calculated by the number of added alleles in the six cryo-bank samples divided by the number of alleles in the 54 contemporary animals from the reference population

### Molecular coancestry

The mean molecular coancestry was 0.416 for pairwise comparisons among all 60 Yakutian cattle individuals (Table 3). The average molecular coancestry calculated between each Yakutian bull and the cows ranged from 0.344 to 0.465. Compared with the living contemporary bull subpopulation, the group of six Yakutian cryo-bank bulls showed a significantly lower (0.395 *vs*. 0.418; a permutation test between the two mean values, *P *= 0.035) mean coancestry with the living contemporary cow subpopulation (Table 3). This indicates that the cryo-bank bulls are good candidates as sires in a breeding program aimed at avoiding inbreeding.

**Table 3:** Average molecular coancestries

Individual	Mean molecular coancestry
Total population	0.42
23 bulls	0.41
17 bulls	0.42
6 bulls	0.40
JA34	0.47
JA32	0.45
JA5	0.44
JA41	0.44
JA42	0.43
JA9	0.43
Alii	0.42
JA38	0.42
JA 17	0.42
JA44	0.42
JA33	0.42
Moxsogol	0.42
JA40	0.41
JA3	0.41
JA43	0.40
Sarial	0.40
Radzu	0.40
JA11	0.40
JA39	0.40
JA31	0.39
Erel	0.38
JA16	0.38
Keskil	0.34

Values are calculated across pairwise comparisons between the individuals in the total population (60 individuals = 6 cryo-bank bulls + 17 contemporary bulls + 37 contemporary cows) and the contemporary 37 cows, between the individuals in the total bull subpopulation (6 cryo-bank bulls + 17 contemporary bulls) and the contemporary 37 cows, between the bulls (17 individuals) and the cows (37 individuals) from the Yakutian reference population, between the six Yakutian cryo-bank bulls and the cows (37 individuals), and between each Yakutian bull and the cows (37 individuals) separately

## Discussion

Knowledge on pairwise relatedness is crucial to draft recommendations for further use of cryo-bank bull semen in conservation and breeding programs of domestic animals. In this study, we have estimated pairwise relatedness among the six Yakutian cryo-bank bulls with different estimators based on autosomal and mtDNA genetic variation. Our study has shown that molecular data provide a useful tool to estimate relatedness among individuals when pedigree data are unavailable. Moreover, the results clearly demonstrate the importance of *ex situ *cryo-banking of genetic material in the conservation of rare domestic animal breeds.

### Relatedness

Our microsatellite analysis suggests that five of the 15 pairwise relatedness comparisons for the Yakutian cryo-bank bulls exhibited coefficients of relatedness (*R*) close to the theoretical expectations for half-sibs (*R *= 25%) and cousins (*R *= 12.5%). However, the two pairwise relatedness estimators identified different Yakutian bull-pairs as clear outliers compared to the simulated distribution of random individuals (Figure 1). Relatedness estimates for simulated unrelated pairs have a very wide distribution: the 95th percentiles (*r*_QG _= 0.242 and *r*_W _= 0.252) are very near or above the theoretical expectation for half-sibs (*R *= 0.25). This indicates that the 30 microsatellite markers used here are insufficient for an unequivocal separation of related and unrelated individuals.

The number and genetic variability of markers as well as population structure might affect the robustness of different methods in the calculation of relatedness between individuals. It has been also demonstrated that there is no single best-performing estimation method to distinguish between all possible types of relatedness [38-40]. In this study, *r*_W _worked better for the simulated categories of related individuals that are important in solving relatedness questions among Yakutian cryo-bank bulls. The approach by [15] is robust for a small sample size and in the cases when the reference population includes unidentified relatives. These assumptions match closely the situation of the Yakutian population studied and therefore could explain the better performance of the estimator.

Thirty markers seems to be sufficient to identify PO's or FS's, but fails to separate HS's or more distant relatedness categories unequivocally. Additional simulations have demonstrated that a set of as many as 500 microsatellites would be needed for much more accurate estimates of *R *with lower standard deviations (results not presented). Our results agree with previous suggestions that a large number of microsatellite loci are needed for unequivocal clarification of pedigrees [33]. Alternatively, using advanced SNP-microchips with thousands of SNP could provide a solution (e.g. [41]).

### Mitochondrial data

MtDNA sequence analysis has shown that the Yakutian cryo-bank bulls do not share any mtDNA haplotype. Nucleotide substitutions accumulate approximately 5 to 10 times faster in mtDNA than in nuclear DNA [42] and cases of mtDNA mutation fixation within one generation have been described in Holstein cattle [43]. However, the smallest pairwise differences between haplotypes observed in Yakutian cryo-bank bulls were two nucleotides. As a result of heteroplasmy, the sons of a dam can have different mtDNA haplotypes. However, no heteroplasmy was detected in the present study. The mtDNA sequence analysis suggests that there are no full-sibs or maternal half-sibs among the Yakutian cryo-bank bulls. Although four Y chromosome-specific microsatellites (*INRA124*, *INRA189*, *BM861 *and *BYM-1*; see [44]) are monomorphic in the population [23], the mean relatedness based on the autosomal microsatellites shows that there are four potential half-sib pairs among the six Yakutian cryo-bank bulls.

### Allelic diversity and gene diversity

The six Yakutian cryo-bank bulls appear to represent an important source of additional allelic variation and gene diversity for the Yakutian bull subpopulation as well as for the total Yakutian population. A high level of genetic diversity would determine the fitness of individuals and would affect the potential response of a population to immediate natural or artificial selection [45].

### Practical recommendations

In a small population, misclassifying related individuals as unrelated (type II error) will result in underestimating relatedness within the population and, thus, represents a risk of increased inbreeding rate in subsequent generations. Therefore, we are more concerned about minimizing the occurrence of type II errors rather than the presence of type I errors, where unrelated individuals are identified as related. In the conservation program for the Yakutian cattle, we recommend that four Yakutian cryo-bank bull-pairs, Keskil:Moxsogol, Radzu:Sarial, Erel:Sarial and also Radzu:Erel, are treated as half-sibs or individuals otherwise having relatedness up to 25%.

In an endangered population, choosing optimal individuals for mating and designing an appropriate mating scheme can help to monitor the genetic variation and the average relatedness among individuals. It has been shown that mating individuals with minimal average coancestries will maximize the population's genetic diversity in terms of expected heterozygosity [46,47]. In our study, 23 Yakutian bulls are candidate sires for the subsequent generation. However, as compared with the contemporary 17 bulls, the six Yakutian cryo-bank bulls show significantly lower average molecular coancestries with the cow population. Using the six cryo-bank semen in artificial insemination would help to control the rate of inbreeding in following generations.

The choice of the mating system is complicated because of the time scale of interest. From a short-term perspective, a simple breeding scheme could be suggested, whereby a population is subdivided into several groups and rotation mating among these groups is performed [48]. In the rotation mating scheme, breeding cows are from the same group as the sire, while breeding bulls are from a different group. Although this scheme will not reduce the degree of inbreeding in the long-run, a more even distribution of inbreeding among individuals would be achieved. Furthermore, it would guarantee that each line produces progeny that will be used for breeding in the next generation. On the basis of the pairwise relatedness among the six Yakutian cryo-bank bulls, we suggest to split them into three separate groups in the rotation mating, with Alii alone in another group, Keskil and Moxogol in one group, and Radzu, Erel and Sarial in a third group.

## Conclusions

With the Yakutian cattle as an example, our results indicate that even a limited number of semen samples selected for the long-term cryo-banking can represent a considerable potential to maintain within-population genetic variability. Therefore, we recommend enrichment of the cryo-bank by adding semen of unrelated bulls with new genetic variability from the current living population. We have shown that when pedigree documentation is unavailable, even a limited number of molecular markers can help to make effective breeding mating schemes, though a larger set of markers would be desirable. We conclude that the present strategy with the help of molecular data can be applied to other animal species or even plants where the reduction of inbreeding and the preservation of genetic variation are important concerns.

## Competing interests

The authors declare that they have no competing interests.

## Authors' contributions

IT designed the study, did the laboratory work, performed the data analysis and drafted the manuscript. MT contributed to the data analysis and the draft writing. MHL contributed to the draft writing and revised the manuscript critically. RP and ZY contributed to the sample collection and the manuscript writing and provided important expertise on Yakutian cattle. JK planned and coordinated the whole study, and contributed to the manuscript writing. All the authors read and approved the final manuscript.

## Supplementary Material

Additional file 1 **Alignment of the variable sites in the 255 nt fragment of the cattle mtDNA control region**. Click here for file

Additional file 2 **Average relatedness estimates for pairwise comparisons among the six Yakutian cryo-bank bulls obtained using relatedness estimators *r*_W _, *r*_QG _and *r*_K_**. Click here for file

Additional file 3 **Mean relatedness and their standard deviations of the two relatedness estimators (*r*_QG _and *r*_W_) for the four simulated relatedness categories**. Click here for file

Additional file 4 **Results of the shared parentage analysis**. Click here for file
