# Supplementary material for: Estimation of relatedness among non-pedigreed Yakutian cryo-bank bulls using molecular data: implications for conservation and breed management
Source: Genet Sel Evol. 2010 Jul 13;42(1):28. doi: 10.1186/1297-9686-42-28 (PMC2909159; doi:10.1186/1297-9686-42-28)
Supplement: Additional file 1 — Alignment of the variable sites in the 255 nt fragment of the cattle mtDNA control region. [file 1297-9686-42-28-S1.DOC]

# Additional files

**Additional file 1 - Alignment of the variable sites in the 255 nt fragment of the cattle mtDNA control region**

Individual names, haplotypes identity (*H*), and the taurine mtDNA haplogroups they belong to are indicated, respectively; identity with consensus sequence (GenBank accession number V00654) is denoted by dots and substitutions by a different base letter; vertical numbers refer to the position of variable site in the 255 nt sequence.

| **Individual** | *H* | **Taurine mtDNA-haplogroup** |  |
| --- | --- | --- | --- |
| Variable site  Consensus  Keskil  Moxsogol  Radzu  Erel  Sarial  Alii  JA50  JA9  JA44  JA5  JA29  JA31  JA20  JA26  JA1  JA10  JA40  JA37  JA18  JA14  JA19  JA38  JA6  JA15  JA34  JA24  JA28  JA33  JA7  JA2 | 1  2  3  4  5  6  7  7  7  7  7  7  7  7  7  7  7  7  7  7  8  8  9  5  10  10  10  10  11  2 | T3  T2  T3  T3  T3  T3  T3  T3  T3  T3  T3  T3  T3  T3  T3  T3  T3  T3  T3  T3  T3  T3  T3  T3  T4  T4  T4  T4  T4  T2 | 111122  22336789999146713  02573380239545515  ATTGAGTCTTTTTGACT  ............C.G..  ...C...T...C.A..C  ..C............T.  ..C.....C........  .C....C..........  ....G............  .................  .................  .................  .................  .................  .................  .................  .................  .................  .................  .................  .................  .................  .................  ..........C......  ..........C......  .........CC......  .C....C..........  .C...A...........  .C...A...........  .C...A...........  .C...A...........  GC...A...........  ...C...T...C.A..C |
