# Supplementary material for: Estimation of relatedness among non-pedigreed Yakutian cryo-bank bulls using molecular data: implications for conservation and breed management
Source: Genet Sel Evol. 2010 Jul 13;42(1):28. doi: 10.1186/1297-9686-42-28 (PMC2909159; doi:10.1186/1297-9686-42-28)
Supplement: Additional file 2 — Average relatedness estimates for pairwise comparisons among the six Yakutian cryo-bank bulls obtained using relatedness estimators rW , rQG and rK. [file 1297-9686-42-28-S2.DOC]

**Additional file 2 - Average relatedness estimates for pairwise comparisons among the six Yakutian cryo-bank bulls obtained using relatedness estimators *r*_W_, *r*_QG_ and *r*_K_**

|  | *r*_W_ | *r*_QG_ | *r*_K_ |
| --- | --- | --- | --- |
| Individual pair |  |  |  |
| Keskil:Moxsogol | 0.242 | 0.295 | 0.320 |
| Keskil:Radzu | -0.263 | -0.149 | 0.000 |
| Keskil:Erel | -0.325 | -0.275 | 0.000 |
| Keskil:Sarial | -0.054 | 0.006 | 0.050 |
| Keskil:Alii | -0.375 | -0.348 | 0.000 |
| Moxsogol:Radzu | -0.011 | 0.021 | 0.016 |
| Moxsogol:Erel | -0.194 | -0.017 | 0.000 |
| Moxsogol:Sarial | -0.075 | -0.033 | 0.000 |
| Moxsogol:Alii | -0.148 | -0.112 | 0.000 |
| Radzu:Erel | 0.170 | 0.255 | 0.160 |
| Radzu:Sarial | 0.276 | 0.195 | 0.230 |
| Radzu:Alii | 0.002 | 0.078 | 0.000 |
| Erel:Sarial | 0.205 | 0.180 | 0.180 |
| Erel:Alii | 0.124 | 0.205 | 0.050 |
| Sarial:Alii | 0.062 | 0.025 | 0.000 |
