# Supplementary material for: Estimation of relatedness among non-pedigreed Yakutian cryo-bank bulls using molecular data: implications for conservation and breed management
Source: Genet Sel Evol. 2010 Jul 13;42(1):28. doi: 10.1186/1297-9686-42-28 (PMC2909159; doi:10.1186/1297-9686-42-28)
Supplement: Additional file 3 — Mean relatedness and their standard deviations of the two relatedness estimators (rQG and rW) for the four simulated relatedness categories. [file 1297-9686-42-28-S3.DOC]

**Additional file 3 - Mean relatedness and their standard deviations of the two relatedness estimators (*r*_QG_ and*r*_W_) for the four simulated relatedness categories**

Unrelated (UR), half-sibs (HS), full-sibs (FS) and parent-offspring (PO) calculated based on allele frequencies from the total Yakutian Cattle population (60 individuals); significance (*P*-values) of the two-tailed *t*-tests for difference between the observed and the expected relatedness values are in parentheses

|  | UR | HS | FS | PO |
| --- | --- | --- | --- | --- |
| *r*_QG_ | 0.004 ± 0.143 (0.196) | 0.256 ± 0.132 (0.152) | 0.505 ± 0.122 (0.170) | 0.512 ± 0.091 (0.0^*^) |
| *r*_W_ | -0.029 ± 0.147 (0.0^*^) | 0.260 ± 0.130 (0.018) | 0.504 ± 0.112 (0.253) | 0.512 ± 0.076 (0.0^*^) |

*Significant after sequential Bonferroni correction (*P* < 0.013; *k* = 4)
