# Supplementary material for: Estimation of relatedness among non-pedigreed Yakutian cryo-bank bulls using molecular data: implications for conservation and breed management
Source: Genet Sel Evol. 2010 Jul 13;42(1):28. doi: 10.1186/1297-9686-42-28 (PMC2909159; doi:10.1186/1297-9686-42-28)
Supplement: Additional file 4 — Results of the shared parentage analysis. [file 1297-9686-42-28-S4.DOC]

**Additional file 4 - Results of the shared parentage analysis**

The upper triangle represents a percentage of all possible of Yakutian bull-pair comparisons (n=2,000) that were identified as full-sibs, the lower triangle represents a percentage of all possible Yakutian bull-pair comparisons (n=2,000) that were identified as half-sibs

|  | Kesil | Moxogol | Radzu | Erel | Sarial | Alii |
| --- | --- | --- | --- | --- | --- | --- |
| Kesil | - | 95.7 % | 0 | 0 | 0 | 0 |
| Moxogol | 100 % | - | 0 | 0 | 0 | 0 |
| Radzu | 0 | 0 | - | 0.75 % | 0 | 0 |
| Erel | 0.6 % | 0.2 % | 81.3 % | - | 23.2 % | 0 |
| Sarial | 35.5 % | 46.65 % | 76.25 % | 96.8 % | - | 0 |
| Alii | 0 | 0 | 15.35 % | 39.8 % | 0.15 % | - |
